# Estrogen receptor alpha and androgen receptor are commonly expressed in well-differentiated liposarcoma

**DOI:** 10.1186/1472-6890-14-42

**Published:** 2014-10-22

**Authors:** Davis R Ingram, Lloye M Dillon, Dina Chelouche Lev, Alexander Lazar, Elizabeth G Demicco, Burton L Eisenberg, Todd W Miller

**Affiliations:** 1 Departments of Surgical Oncology, M.D. Anderson Cancer Center, University of Texas, 1515 Holcombe Blvd, Houston, TX, USA; 2 Departments of Pharmacology & Toxicology, Norris Cotton Cancer Center, Geisel School of Medicine at Dartmouth, Dartmouth-Hitchcock Medical Center, One Medical Center Drive, HB-7936, Lebanon, NH 03756, USA; 3 Departments of Surgical Pathology, M.D. Anderson Cancer Center, University of Texas, 1515 Holcombe Blvd, Houston, TX, USA; 4 Departments of Surgery, Norris Cotton Cancer Center, Geisel School of Medicine at Dartmouth, Dartmouth-Hitchcock Medical Center, One Medical Center Dr, Lebanon, NH, USA; 5 Department of Pathology, Mount Sinai Medical Center, One Gustave L. Levy Pl, New York, NY, USA

## Abstract

**Background:**

Liposarcoma (LS) is the second-most common type of soft-tissue sarcoma. Despite advances in knowledge and treatment of this disease, there remains a need for more effective LS therapy. Steroid hormone receptors regulate metabolism in adipocytes. Estrogen receptor alpha (ER), progesterone receptor (PR), and androgen receptor (AR) have been implicated in the pathophysiology of other cancer types. We sought to comprehensively determine temporal expression patterns of these receptors in LS.

**Methods:**

We analyzed 561 histologically subtyped LS specimens from 354 patients for expression of ER, PR, and AR by immunohistochemistry (IHC) using diagnostic-grade reagents and protocols. The fractions of positively stained tumor cells were scored within each specimen. IHC scores were compared across LS subtypes using the Kruskal-Wallis test, and subtypes were compared using Dunn’s post-hoc test. Ages of patients with receptor-positive vs. -negative LS were compared by t-test. Genders and races were compared for hormone receptor positivity using Fisher’s exact test and Chi-square analysis, respectively. Recurrence-free survival was compared between receptor-positive and negative patients by log-rank test. p< 0.05 was considered significant.

**Results:**

ER and AR were frequently expressed in LS, while few tumors expressed PR. Most of the ER + and AR + samples were of the well-differentiated LS subtype. A smaller fraction of de-differentiated LS expressed ER or AR, but expression was common within well-differentiated regions of tumors histologically classified as de-differentiated LS. In LS specimens from patients who underwent multiple surgeries over time, receptor expression frequently changed over time, which may be attributable in part to intratumor heterogeneity, varying degrees of de-differentiation, and biopsy bias. ER and AR were frequently co-expressed. Receptor status was not significantly associated with gender or race, but AR and PR expression were associated with earlier age at diagnosis. Receptor expression was not associated with altered recurrence-free survival.

**Conclusions:**

ER and AR are commonly expressed in LS, particularly in well-differentiated tumors. These data warrant further functional study to determine receptor function in LS, and the potential efficacy of anti-hormone therapies for the treatment of patients with LS.

## Background

Approximately 11,280 patients are diagnosed with one of many types of soft tissue sarcoma each year in the U.S. [1]. Liposarcomas (LS) constitute approximately 24% of extremity and 45% of retroperitoneal soft tissue sarcomas [2], ranking as the second-most common type of soft-tissue sarcoma. LS occurs in three major biologic subgroups: 1) well- or de-differentiated LS (WDLS, DDLS, most common subgroup), 2) myxoid LS (MLS), and 3) pleomorphic LS (PLS). WDLS and MLS are typically low-grade tumors, DDLS are often intermediate grade with intermediate risk for metastasis, and PLS are high-grade and clinically aggressive. It is thought that DDLS starts as WDLS, and tumor cells progressively accumulate genetic lesions as they transition to a less differentiated, non-lipogenic state. Progression to DDLS is associated with more aggressive local disease, increased metastatic potential (10-20%), and increased mortality (50-75%) [3-6]. LS is typically treated by surgical resection, and high-grade lesions are sometimes treated with adjuvant radiation therapy. DNA-damaging chemotherapy is usually not effective against LS. In addition, tumor recurrence is common, particularly with retroperitoneal LS. Therefore, there exists a need for improved LS therapy.

LS likely originates from a lipogenic precursor cell(s). Since lipogenic metabolism is heavily influenced by steroid hormones [7], and adipocytes express nuclear hormone receptors and steroidogenic enzymes such as aromatase [8,9], we postulated that LS cells may similarly express such receptors. Prior studies in limited numbers of patients reported expression of steroid hormone receptors in a fraction of LS cases [10-17]. To expand upon and clarify these findings, we analyzed 561 LS specimens acquired from 353 patients in the largest LS cohort reported to-date to determine the frequencies of expression of estrogen receptor alpha (ER), progesterone receptor (PR), and androgen receptor (AR). Frequent expression of these hormone receptors may prompt clinical testing of anti-hormone strategies, such as those used to treat patients with cancers of the breast (anti-estrogens) or prostate (anti-androgens), or to control pregnancy (anti-progestins), in order to assess the contribution of these receptors to LS growth. These drugs may ultimately prove useful for the treatment of patients with LS.

## Methods

### Patients and tissues

LS specimens were obtained at Dartmouth-Hitchcock Medical Center and M.D. Anderson Cancer Center between 1986 and 2012 under protocols approved by the Institutional Review Boards of Dartmouth-Hitchcock Medical Center and M.D. Anderson Cancer Center, respectively. Patients provided written informed consent. Tissues were formalin-fixed and paraffin-embedded. Core samples were used to construct tissue microarrays (TMAs). Clinical records indicated that these TMAs included 379 tumors from 353 patients, where tumors were classified as DDLS (*n=* 122), WDLS (*n=* 146), MLS (*n=* 79), or PLS (*n=* 32). WDLS and DDLS are thought to represent different stages of disease progression that can co-exist in the same tumor. Hence, some cores taken from DDLS cases were histologically classified as “WDLS.” In total, we analyzed 561 core samples, which were classified as 294 WDLS, 123 DDLS, 112 MLS, and 32 PLS based on histological criteria.

### Immunohistochemistry

Commercially available antibodies against ER alpha (6 F11 monoclonal, dil. 1:35; Leica Biosystems), PR (PgR 1294 monoclonal, dil. 1:200; Dako; recognizes both A and B isoforms), and AR (AR441 monoclonal, dil. 1:30; Dako; recognizes both A and B isoforms) were used for immunohistochemistry (IHC). These antibodies are routinely used for *in vitro* diagnostics in clinical laboratories. A Leica BOND-MAX automated stainer was used with a polymer/HRP detection system. After deparaffinization, 5-μm TMA sections were treated with citrate buffer at 100°C for 25–30 minutes. Slides were probed with primary antibody for 15 minutes, washed, and probed with HRP-polymer anti-mouse IgG for 8 minutes. Signal was detected using 3,3-diaminobenzidine, followed by hematoxylin counterstaining. ER+/PR + breast tumor tissue was used as a positive control for ER and PR IHC. AR + prostate cancer tissue was used as a positive control for AR IHC. Spleen biopsies were included in the TMAs and used as negative control tissues. Tissues were scored based on the estimated percent of positively stained cancer cell nuclei.

### Statistics

IHC scores were compared across LS subtypes using the Kruskal-Wallis non-parametric test, and subtypes were compared in a one-by-one fashion using Dunn’s post-hoc test. Ages of patients with hormone receptor-positive vs. -negative WDLS/DDLS were compared by *t*-test. Genders and races were compared for hormone receptor positivity using Fisher’s exact test and Chi-square analysis, respectively. *p* ≤ 0.05 was considered significant.

## Results

### Frequent expression of ER and AR in WDLS and DDLS

**Figure 1:**
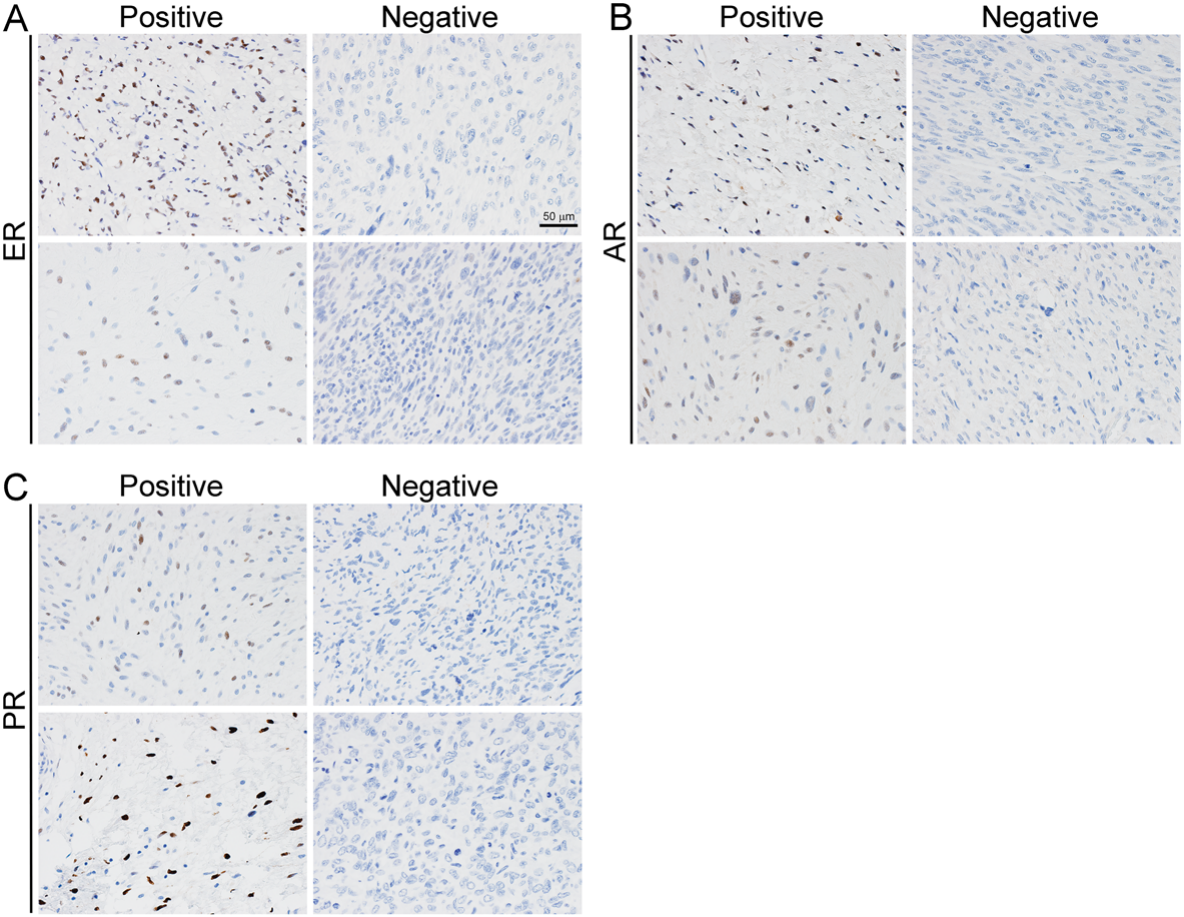
**Steroid hormone receptor expression in LS.** Sections of LS were stained using antibodies against **A)** ER, **B)** AR, or **C)** PR. Shown are two representative microscopic fields that were scored as receptor-positive or -negative. Scale bar in **(A)** is 50 μm.

**Figure 2:**
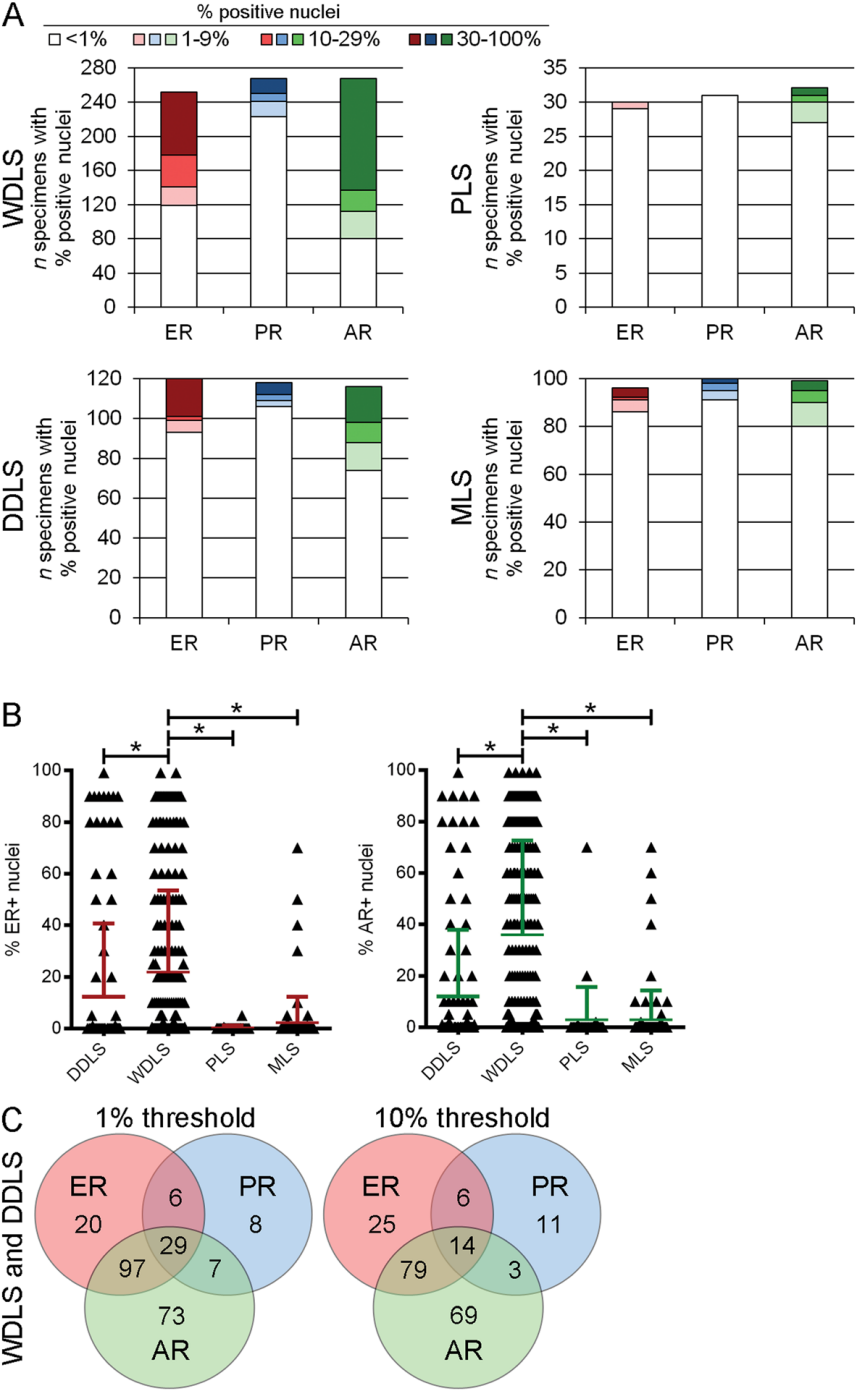
**Steroid hormone receptor expression is most common in WDLS. A)** LS specimens were scored for% positively-stained nuclei for ER, PR, and AR, then classified by histological subtype and binned according to score as indicated. **B)** ER and AR scores were compared between specimen subtypes. Colored bars indicate mean + SD. *p< 0.0001 by Dunn’s post-hoc test. **C)** Venn diagrams illustrating the number of WDLS and DDLS specimens with co-expression of hormone receptors using a threshold of 1% or 10% positively-stained nuclei.

IHC staining of 561 specimens obtained from 379 LS tumors from 353 patients (characteristics listed in Table 1) revealed nuclear ER and AR expression in a significant number of cases (Figure 1). PR staining was less frequently observed. Using the stringent threshold of 10% positively-stained nuclei, we observed the following: 43.1% of WDLS, 17.5% of DDLS, 0% of PLS, and 5.2% of MLS were scored as ER+; 9.8% of WDLS, 7.6% of DDLS, 0% of PLS, and 5% of MLS were scored as PR+; 58.1% of WDLS, 24.1% of DDLS, 6.2% of PLS, and 9.1% of MLS were scored as AR + (Figure 2A). Using the threshold of 1% positively-stained nuclei used in the histological classification of breast cancer, we observed the following: 52.8% of WDLS, 22.5% of DDLS, 3.3% of PLS, and 10.4% of MLS were scored as ER+; 16.5% of WDLS, 10.2% of DDLS, 0% of PLS, and 9% of MLS were scored as PR+; 70% of WDLS, 36.2% of DDLS, 15.6% of PLS, and 19.2% of MLS were scored as AR + .

**Table 1:** Baseline characteristics

**Characteristic**	***n *** **patients**
**Age at registration**	
<30 years	9
30–50 years	106
50.1-70 years	180
>70 years	51
Unreported	7
**Gender**	
Male	215
Female	138
**Race**	
White	264
Black	12
Hispanic	38
Asian	7
Unreported	32
**Primary tumor type**	
Well-differentiated	136
De-differentiated	107
Myxoid	78
Pleomorphic	32
**Primary tumor size**	
<5 cm	15
5–9.9 cm	55
≥10 cm	221
Undetermined	62
**Chemotherapy**	
Yes	98
No	236
Unreported	19
**Radiation therapy**	
Yes	93
No	239
Unreported	21

Since a significant number of specimens expressed ER or AR, we compared IHC scores between histologic subtypes. We detected a statistically significantly higher frequency of ER and AR expression in WDLS compared to each other subtype (Figure 2B, all *p<* 0.001). While some DDLS specimens expressed ER and AR, this subtype was not significantly different from PLS or MLS. PR was not significantly differentially expressed across histologic subtypes.

We then used Venn diagrams to determine the frequency of receptor co-expression within the combined WDLS and DDLS subtypes. Among 347 WDLS/DDLS specimens for which ER and AR IHC were evaluable, ER and AR were co-expressed in 28% and 22.8% of specimens at the 10% and 1% thresholds, respectively (Figure 2C).

### Intratumor heterogeneity in ER and AR expression

**Figure 3:**
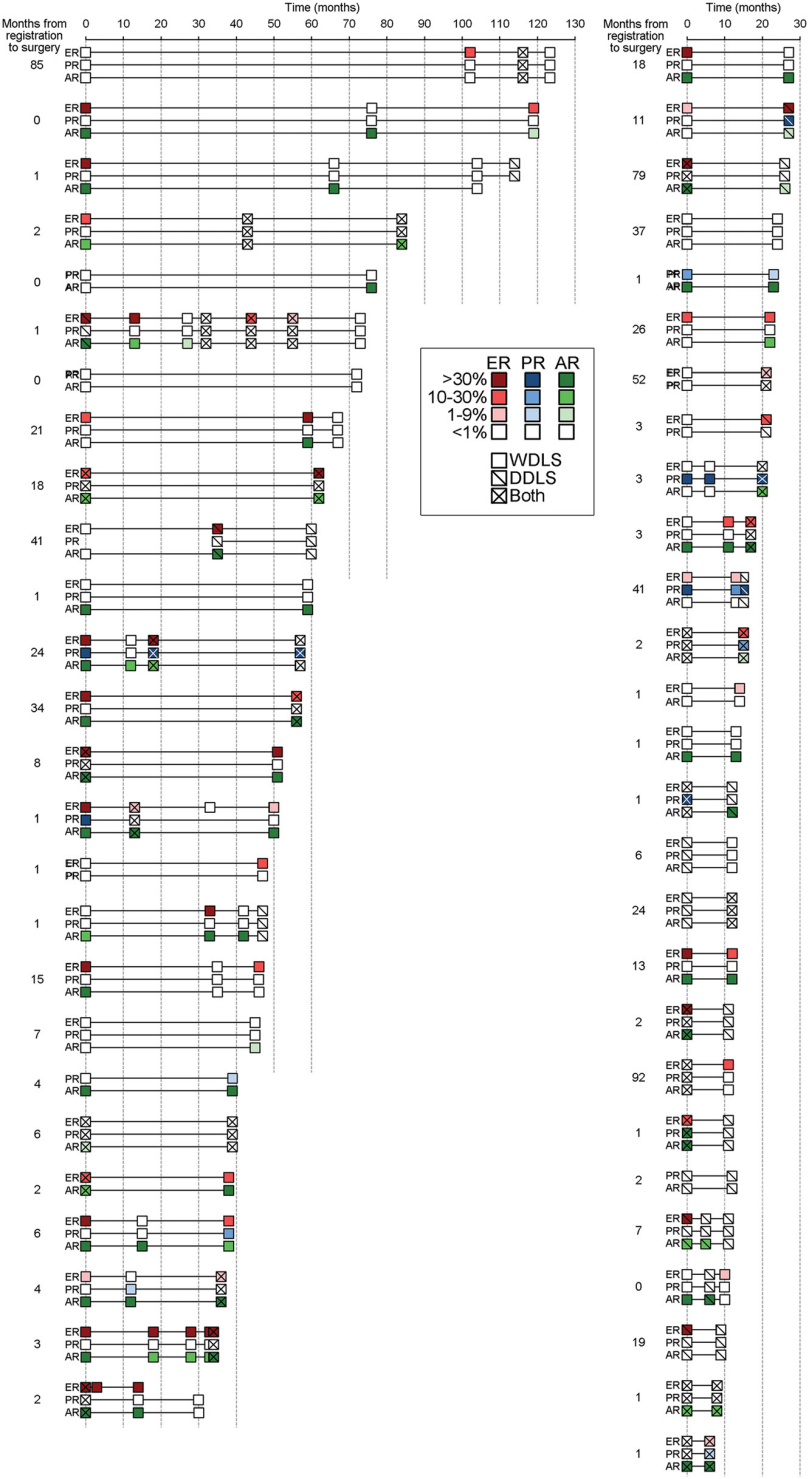
**Changes in tumor hormone receptor status over time.** Fifty-three patients with WDLS and/or DDLS for whom specimens were obtained from ≥2 surgeries ≥6 months apart were evaluated. In cases where multiple specimens were obtained from the same tumor at the same time point, the% positively-stained nuclei were averaged across specimens. The% positively-stained nuclei and histological classification of each tumor are noted by color intensity and hash marks as indicated in legend.

We observed that hormone receptor expression sometimes differed between tumors acquired from the same patient who underwent multiple surgeries over time. We therefore systematically evaluated hormone receptor expression levels over time in 53 patients who underwent ≥2 surgeries to remove WDLS and/or DDLS (Figure 3). This analysis revealed that many tumors show changes in hormone receptor expression over time, which may be attributable in part to intratumor heterogeneity, varying degrees of de-differentiation, and biopsy bias.

**Figure 4:**
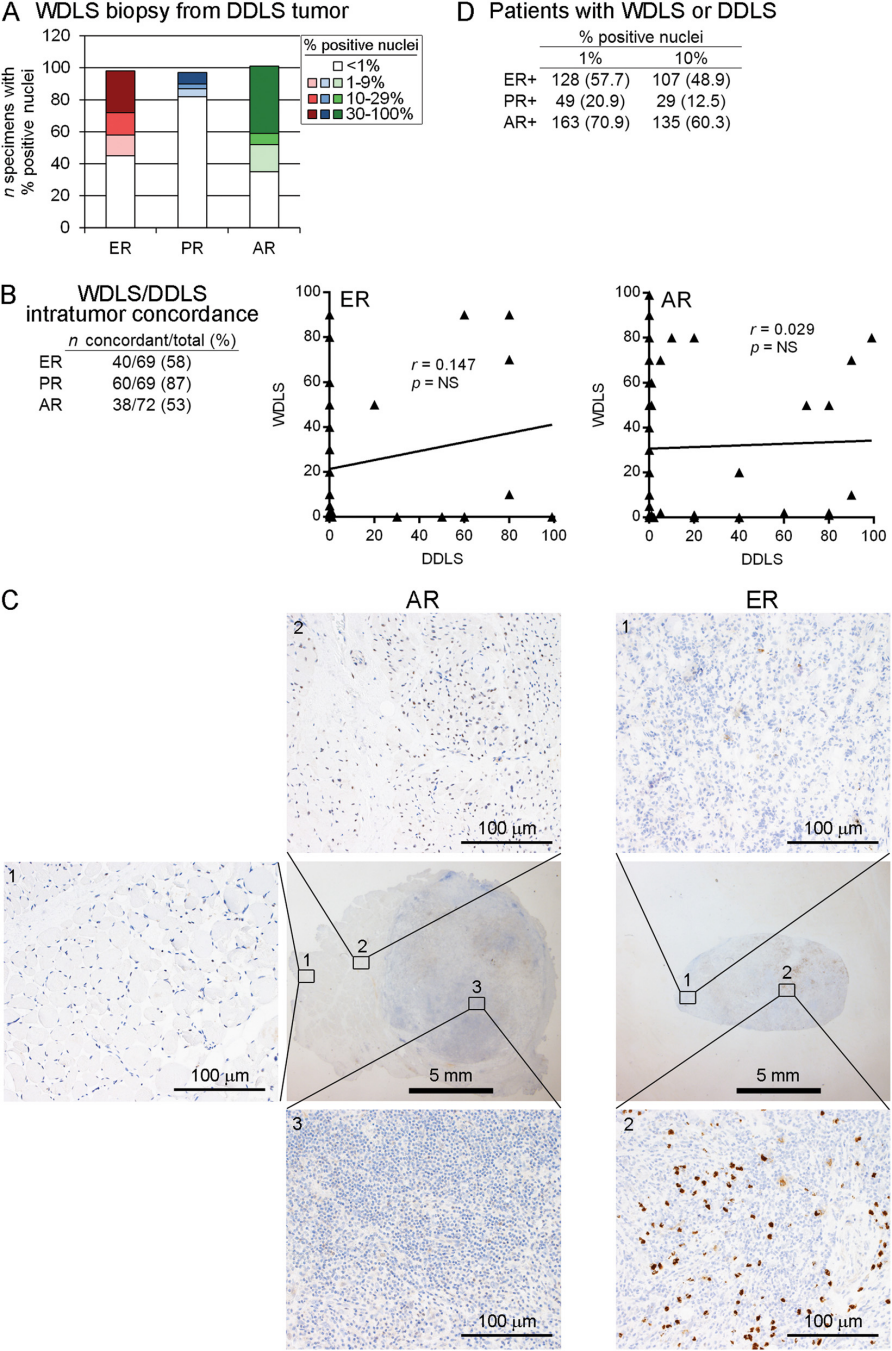
**Intratumor heterogeneity in degree of differentiation contributes to heterogeneity in hormone receptor expression. A)** Frequencies of hormone receptor expression among WDLS specimens obtained from tumors classified as DDLS. **B)** Tumors for which 2 specimens were obtained from different regions at the same time point were evaluated for concordance in hormone receptor expression using a threshold of 10% for positivity. Scatterplots show% positive nuclei for ER and AR in tumors for which one DDLS specimen and one WDLS specimen were available. **C)** Representative liposarcoma specimens showing regions of receptor-positivity and -negativity. **D)** Hormone receptor status for patients with WDLS/DDLS as determined using any LS specimen obtained from any surgery.

DDLS represents a form of tumor progression in WDLS, and both histologies may co-exist within the same tumor. We evaluated DDLS tumors with synchronous foci of WDLS and DDLS histologies for hormone receptor expression. At a threshold of 10% positively-stained nuclei, 40.8%, 10.3%, and 48.5% of WDLS foci were ER+, PR+, and AR+, respectively. At a 1% threshold, 54.1%, 15.5%, and 65.3% of WDLS foci were ER+, PR+, and AR+, respectively (Figure 4A). Therefore, ER and AR expression are common in WDLS foci within otherwise DDLS tumors.

We then determined the rate of concordance for hormone receptor expression between 2 specimens obtained from different regions of the same WDLS/DDLS tumor; 75 tumors were available for this analysis. Using a threshold of 10% positively-stained nuclei, this analysis revealed rates of 58% and 53% concordance for ER and AR, respectively (Figure 4B). Ninety-two percent (69/75) of these tumors had specimens histologically classified as WDLS and DDLS (one of each). Among the ER-discordant tumors, 82.8% (24/29) showed an ER + WDLS specimen and an ER- DDLS specimen (Figure 4B). Among the AR-discordant tumors, 73.7% (28/38) showed an AR + WDLS specimen and an AR- DDLS specimen. These data suggest that the high degree of intratumor discordance in ER and AR expression (demonstrated with representative specimens in Figure 4C) is partially attributable to the degree of de-differentiation.

Given that tumor hormone receptor status changed over time (Figure 3), we evaluated associations between patient characteristics and hormone receptor expression using hormone receptor status (threshold of 10% positively-stained nuclei) determined from A) a specimen obtained from the first surgery performed at M.D. Anderson Cancer Center, or B) hormone receptor positivity from a specimen obtained from any WDLS/DDLS surgical specimen (*i.e.*, patient had a receptor-positive specimen at any time point; frequencies shown in Figure 4D). The latter criterion indicated that 48.9% and 60.3% of patients had ER + or AR + WDLS/DDLS at some point during the course of their disease. Hormone receptor status was not significantly associated with gender, race, or tumor size. There was a significant association linking AR expression with earlier age at WDLS/DDLS diagnosis, and a trend linking PR expression with earlier age at diagnosis (Tables 2 and 3). However, the mean ages of onset were similar between receptor-positive and -negative groups. ER and AR expression were not associated with recurrence-free survival in patients with WDLS and/or DDLS using either criterion.

**Table 2:** Receptor positivity at any time point

					**Gender**		
		***n***	**Age (years)**	**t-test p**	**Male**	**Female**	**Fisher's p**
ER	Negative	117	60.6 ± 11.8		74	43	
	Positive	102	59.6 ± 11.1	NS	57	45	NS
	Undetermined	24			13	11	
	Total	243			144	99	
PR	Negative	203	60.4 ± 11.3		121	82	
	Positive	28	56.4 ± 13.2	0.08	14	14	NS
	Undetermined	12			9	3	
	Total	243			144	99	
AR	Negative	90	61.3 ± 11.8		52	38	
	Positive	134	58.6 ± 11.4	0.09	83	51	NS
	Undetermined	19			9	10	
	Total	243			144	99	

**Table 3:** Receptor positivity at time of first surgery

					**Gender**		
		***n***	**Age (years)**	**t-test p**	**Male**	**Female**	**Fisher's p**
ER	Negative	124	60.9 ± 11.3		80	44	
	Positive	90	59.1 ± 11.6	NS	50	40	NS
	Undetermined	29			14	15	
	Total	243			144	99	
PR	Negative	202	60.4 ± 11.3		122	80	
	Positive	23	55.5 ± 13.7	0.06	11	12	NS
	Undetermined	18			11	7	
	Total	243			144	99	
AR	Negative	100	61.6 ± 11.6		58	42	
	Positive	122	58.0 ± 11.5	0.02	76	46	NS
	Undetermined	21			10	11	
	Total	243			144	99	

## Discussion

Given that WDLS have a significantly higher frequency of ER-positivity and AR-positivity than DDLS, and that WDLS foci within DDLS tumors are often ER + and/or AR+, hormone receptor expression is likely associated with a more differentiated LS phenotype. WDLS is often locally aggressive and non-metastasizing, is treated with surgical resection, and occurs repeatedly particularly in the retroperitoneum or mediastinum. WDLS causes morbidity through uncontrolled local effects on vital organs, or through de-differentiation and metastasis. Therefore, therapeutics to control WDLS and prevent de-differentiation may be clinically valuable.

If ER and AR are functionally important for WDLS/DDLS cell proliferation or viability, as is observed in other cancer types such as breast and prostate, anti-hormone therapies may prevent LS progression. However, we caution that hormone receptor expression does not necessarily indicate receptor dependence. AR is expressed in 70-90% of breast cancers [18], but clinical testing of anti-androgen therapy in unselected patients with breast cancer met with little success [19]. AR may be functionally important in certain breast cancer subtypes [20,21], and clinical testing of anti-androgen therapy in patients with such subtypes is ongoing. Furthermore, ER + breast tumors frequently co-express PR. Since PR is encoded by an ER-regulated gene, PR co-expression is typically indicative of ER function. Surprisingly, most ER + LS specimens are PR-, raising the possibility that ER is non-functional. Alternatively, ER may regulate a different set of genes in LS cells, and/or PR levels may be modulated by another mechanism (such as phosphorylation by mitogen-activated protein kinase (MAPK), which promotes degradation [22]).

*In vitro* evidence to support hormone receptor dependence in LS models would help elucidate receptor function, but few WDLS cell lines exist, and it is arguable whether such cell lines accurately model the disease(s). Transgenic mice that overexpress *IL22* in adipocytes develop WDLS when fed a high-fat diet [23]. If such murine LS tumors are hormone receptor-positive, this may present a useful model to elucidate receptor functionality in LS. There have been no proof-of-principle clinical trials to evaluate the effects of anti-hormone therapies in LS based on pre-treatment tumor hormone receptor status. Another option to explore the role(s) of hormone receptors in LS would be a pilot presurgical clinical study, where patients who have undergone a diagnostic tumor biopsy would be treated with anti-hormone therapy for 2–3 weeks prior to surgical tumor resection. The diagnostic (pre-treatment) biopsy tissue is then compared to the surgical (post-treatment) specimen to determine whether levels of hormone receptor-driven or cell cycle-related genes and proteins have changed. This strategy may be useful to identify LS patients who will (or will not) benefit from adjuvant endocrine therapy [24].

## Conclusions

In summary, we demonstrate that a significant fraction of WDLS and DDLS express ER and/or AR. The hormone receptor scoring method used herein is not finely calibrated, which may affect assay sensitivity; however, a less sensitive scoring method may be more likely to detect only cases with more robust (and, likely, more biological important) expression of these receptors. While there appears to be intratumor heterogeneity in hormone receptor expression, both between well- and de-differentiated areas of the same tumor, and over time within a patient’s tumor (which may be partially attributable to biopsy bias), endocrine therapeutics may be useful to control hormone receptor-driven LS cells and mitigate disease progression.

## Abbreviations

ER: Estrogen receptor alpha; PR: Progesterone receptor; AR: Androgen receptor; LS: Liposarcoma; WDLS: Well-differentiated liposarcoma; DDLS: De-differentiated liposarcoma; MLS: Myxoid liposarcoma; PLS: Pleomorphic liposarcoma.

## Competing interests

The authors declare that they have no competing interests.

## Authors’ contributions

TWM and DCL designed the study. DR, DCL, AL, EGD, and TWM procured tissue samples, generated the tissue microarray, and performed IHC staining. TWM, LD, and BLG scored IHC staining and analyzed data. TWM wrote the manuscript. All authors read, provided input on, and approved the final manuscript.

## Pre-publication history

The pre-publication history for this paper can be accessed here:

http://www.biomedcentral.com/1472-6890/14/42/prepub
